# Truncated DAZL mutation reduces NANOS3 expression in primordial germ cells and leads to premature ovarian insufficiency

**DOI:** 10.1093/lifemedi/lnae007

**Published:** 2024-02-09

**Authors:** Qingyuan Liu, Ting Guo, An Yan, Kehkooi Kee

**Affiliations:** The State Key Laboratory for Complex Severe and Rare Diseases, SXMU-Tsinghua Collaborative Innovation Center for Frontier Medicine, Department of Basic Medical Sciences, School of Medicine, Tsinghua University, Beijing 100084, China; Center for Reproductive Medicine, Cheeloo College of Medicine, Shandong University, Jinan 250012, China; Key Laboratory of Reproductive Endocrinology of Ministry of Education, National Research Center for Assisted Reproductive Technology and Reproductive Genetics, Shandong Key Laboratory of Reproductive Medicine, Shandong Provincial Clinical Research Center for Reproductive Health, Jinan 250012, China; The State Key Laboratory for Complex Severe and Rare Diseases, SXMU-Tsinghua Collaborative Innovation Center for Frontier Medicine, Department of Basic Medical Sciences, School of Medicine, Tsinghua University, Beijing 100084, China; The State Key Laboratory for Complex Severe and Rare Diseases, SXMU-Tsinghua Collaborative Innovation Center for Frontier Medicine, Department of Basic Medical Sciences, School of Medicine, Tsinghua University, Beijing 100084, China

**Dear Editor,**

Premature ovarian insufficiency (POI) is defined as the dysfunction or deficiency of ovarian follicles in women younger than 40 years old [1]. Several factors, including genetic, infectious, metabolic, and autoimmune causes, have been identified as contributing to POI [2]. Deleted in *Azoospermia-like* (*DAZL*, or *Dazla)*, a germ line-specific gene, encodes an RNA-binding protein that plays a crucial role in multiple stages of germ cell development. These stages include the late primordial germ cells (PGCs) to meiosis initiation, spermatogenesis, and oogenesis [3]. During early to late PGC transition, DAZL functions to down-regulate the expression of pluripotency genes and modulates the proliferation of PGCs through enhancing specific miRNA maturations in human, indicating the vital role of DAZL in human gametogenesis [4, 5]. At the molecular level, DAZL engages in interactions with multiple mRNA transcripts through its RNA recognition motif, enabling binding to the 3ʹ-untranslated region (UTR) [6]. In order to facilitate the translation of mRNA, DAZL protein recruits poly(A)-binding proteins (PABPs) and promotes the assembly of 80S ribosomes [7]. Notably, several missense mutations in DAZL have been identified in women suffering premature ovarian failure or spontaneous early menopause [8]. However, no truncated mutation has been reported in DAZL that contributes to human ovarian failure.

In this study, whole-exome sequencing was performed on DNA sample of a patient diagnosed with POI, and a homozygous point mutation in the *DAZL* gene was identified. The mutation, c.808C >T, is located at chr3:16633646 (Table S1). This variant is absent from the gnomAD (www.gnomad-sg.org) and 1000 Genomes database. Pathogenicity annotation analyses (combined annotation-dependent depletion (CADD) and deleterious annotation of genetic variants using neural networks (DANN)) revealed that this variant was likely to be deleterious, with a CADD score of 13.086 and a DANN score of 0.996 (Table S1). The c.808C>T variant introduced a premature termination codon at amino acid 270, generating a truncated protein variant, p.Arg270*, which lacked 135 bp on the 3ʹ-end, including the entire exon 11, or 45 amino acids at the C-terminal end of DAZL (Fig. 1A and 1B). The amino acids alignment analysis showed that Arg270 and the neighboring amino acids are highly conserved among many species, suggesting that the substitution of arginine with a stop codon could have deleterious effects (Fig. 1D).

**Figure 1.**
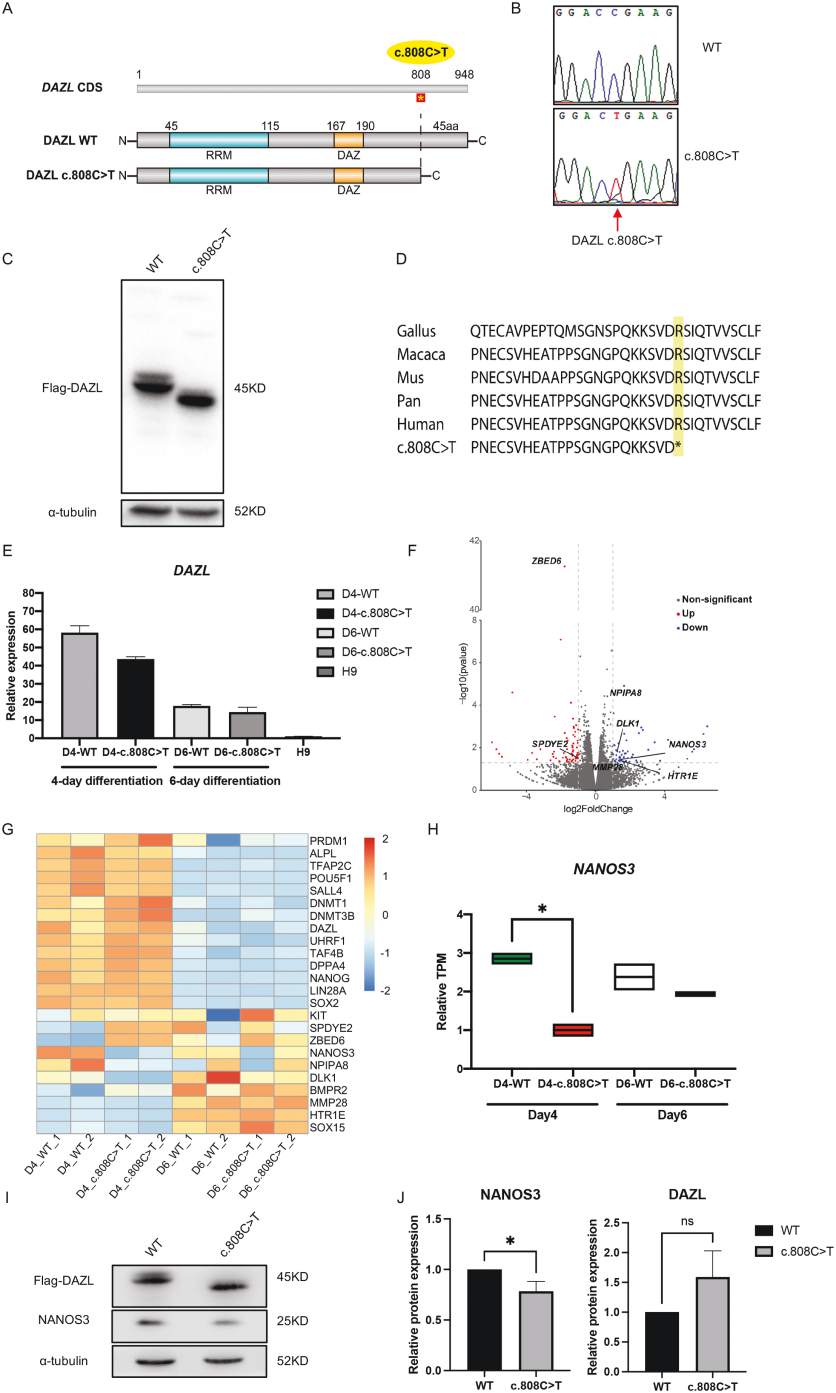
**A homozygous point mutation of DAZL gene was identified in a premature ovarian insufficiency patient, causing a down-regulation of germline gene *NANOS3* in hPGCLCs.**(A) The mutated site was showed in DAZL coding sequence (CDS) and DAZL protein. The full length of DAZL CDS was 948 bp, the variant site was c.808C>T (yellow round). The c.808C>T variant generated a premature termination codon at amino acid 270. (B) Validation of the homozygous point mutation of DAZL gene. Sanger sequencing result was shown. Red arrow indicates the c.808C>T variant site. (C) Western blot analysis of Flag-tagged DAZL expression. H9 cells were transduced with lentivirus carrying mCherry-P2A-3×Flag-wildtype DAZL (WT) and c.808C>T DAZL (c.808C>T). α-Tubulin was used as internal control. (D) The variant site was conserved among multiple species. (E) RT-qPCR analysis of DAZL mRNA level in hES H9 cells, 4-day and 6-day differentiated hPGCLCs overexpressing WT or c.808C >T DAZL (*n* = 4). Ct values were normalized to GAPDH. (F) Volcano plot of differentially expressed genes between 4-day differentiated hPGCLCs overexpressing WT and c.808C>T DAZL. (G) Heatmap of the expression levels of selected germ lineage genes between 4-day differentiated hPGCLCs overexpressing WT and c.808C>T DAZL. (H) Relative TPM of NANOS3 in 4-day and 6-day differentiated hPGCLCs overexpressing WT and c.808C>T DAZL (*n* = 2). *, *P* < 0.05. Two-tailed Student’s *t*-test. (I) Western blot analysis of NANOS3 expression levels in 4-day differentiated hPGCLCs overexpressing WT and c.808C>T DAZL. α-Tubulin was used as internal control. (J) Relative NANOS3 and DAZL protein expression were analyzed by ImageJ software (*n* = 3). Integrated density values were normalized to α-tubulin density values. *, *P* < 0.05. Two-tailed Student’s *t*-test.

To investigate the functional consequences of the c.808C >T mutation on *DAZL*, we introduced this point mutation into the human *DAZL* sequence (c.808C>T *DAZL*) and examined its expression in human embryonic stem cell (hESC) H9 line. Western blot analysis confirmed that the mutant DAZL protein was slightly smaller than wild-type (WT) DAZL (5.536 kDa according to the molecular weight of the truncated peptide), but the mutant DAZL exhibited similar expression level to the WT DAZL protein (Fig. 1C).

To examine the potential effect of the mutant DAZL on PGC gene expressions, we performed transcriptome analysis using *in vitro* differentiated human PGC-like cells (hPGCLCs). WT DAZL or the truncated DAZL was overexpressed to a similar level in hPGCLCs during *in vitro* differentiation for 4 or 6 days (Fig. 1E). The volcano plot illustrated differential expression of 57 genes between 4-day hPGCLCs containing WT DAZL and the mutant DAZL, with 31 genes being up-regulated and 26 genes being down-regulated (Fig. 1F). Heatmap analysis of selected genes revealed distinct expression pattern in subset of the genes in WT versus mutant DAZL hPGCLCs (Fig. 1G). Pluripotency marker genes, including *SOX2*, *NANOG*, *LIN28A*, *SALL4*, and *POU5F1*, were expressed higher in 4-day versus 6-day hPGCLCs. Conversely, some PGC marker genes such as *SOX15* showed higher expression levels in 6-day differentiated hPGCLCs compared to the 4-day hPGCLCs. Notably, 4-day WT hPGCLCs expressed higher level of *NANOS3* (Fig. 1G and 1H), among other genes, than the hPGCLCs expressing mutant DAZL, indicating that the truncated DAZL may contribute to a defect in NANOS3 expression. Consistently, Western blot analysis revealed a notable decrease in NANOS3 expression specifically in c.808C>T DAZL hPGCLCs (Fig. 1I and 1J). These results indicated that the truncated DAZL led to lower NANOS3 expression compared with WT DAZL in hPGCLCs.

DAZL has been reported to regulate multiple genes including late PGCs marker genes *VASA* and *SYCP3* via post-transcriptional regulation at their 3ʹUTRs [4]. To address this, we performed 3ʹUTR dual luciferase reporter assays to investigate whether truncated DAZL may have defect on regulating the 3ʹUTRs of its target genes. As expected, luciferase activities of NANOS3, VASA, and SYCP3 were significantly higher in WT DAZL overexpression groups compared to the control groups (Fig. 2A). In contrast, the truncated DAZL overexpression groups exhibited significantly lower luciferase activities of NANOS3, VASA, and SYCP3 compared to the WT DAZL. These results indicated that the c.808C>T mutation in DAZL displayed defects in the post-transcriptional regulation of germ-line marker genes *NANOS3*, *VASA*, and *SYCP3*. Previous studies have demonstrated that DAZL can bind to the 3ʹUTR and recruit poly(A)-binding protein (PABP) through protein-protein interaction, resulting in a closed-loop conformation and enhanced ribosome recruitment [7]. We sought to investigate whether the truncated DAZL exerts a detrimental effect on its interaction with the co-factor PABP, potentially resulting in impaired post-transcriptional regulation. The co-immunoprecipitation (co-IP) experiments were performed using antibodies against PABP or Flag-tagged DAZL in HEK293FT cells overexpressing WT DAZL or the truncated DAZL. As expected, we detected the presence of Flag-tagged DAZL and PABP in the precipitated products of the WT and c.808C>T groups. However, the level of PABP co-IP with the truncated DAZL was significantly lower than with the WT DAZL (Figs. 2B and S1). The co-IP analysis confirmed that WT DAZL could efficiently interact with the co-factor PABP, while the truncated DAZL exhibited a defect in its interaction with PABP. Taken together, these results suggested that the truncated DAZL exhibited reduced efficiency in recruiting PABP, resulting in the lower post-transcriptional expression of its target mRNAs.

**Figure 2.**
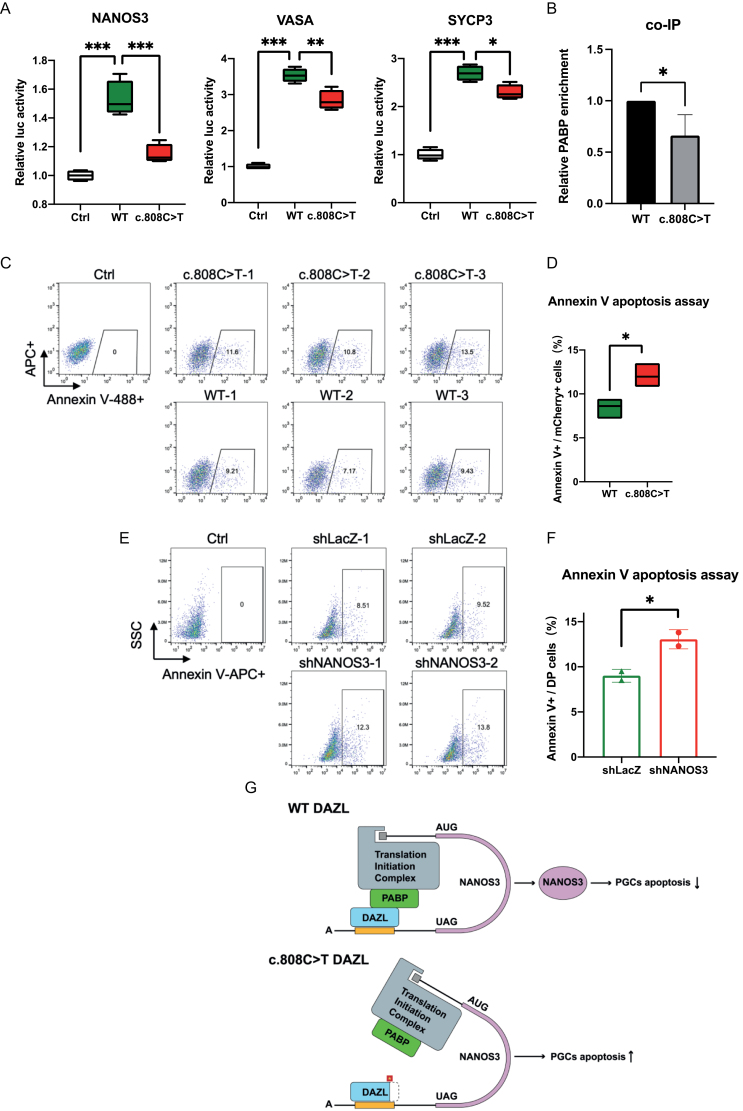
**The c.808C>T variant exhibited defects on translational regulations and promoted cell apoptosis in hPGCLCs.**(A) 3ʹUTR dual luciferase reporter assays of NANOS3, VASA and SYCP3 in HEK293FT cells overexpressing empty vector (Ctrl), WT or c.808C >T DAZL (*n* = 4). ***, *P* < 0.001, **, *P* < 0.01, *, *P* < 0.05. Statistical significances were calculated using one-way ANOVA. (B) The interaction between PABP and WT or c.808C>T DAZL was validated by co-immunoprecipitation (Fig. S1). Relative PABP protein enrichment was analyzed by ImageJ software (*n* = 3). Integrated density values were normalized to Flag density values. *, *P* < 0.05. Two-tailed Student’s *t*-test. (C) Flow cytometry analysis of cell apoptosis in hPGCLCs overexpressing WT or c.808C>T DAZL stained with Annexin V Alexa Fluor488. APC was an irrelevant channel to exclude spontaneous fluorescence. (D) The Annexin V Alexa Fluor488-positive cells proportion of mCherry positive hPGCLCs (*n* = 3). *, *P* < 0.05. Two-tailed Student’s *t*-test. (E) Flow cytometry analysis of cell apoptosis in NANOS3 knockdown WT hPGCLCs stained with Annexin V Alexa Fluor647. (F) The Annexin V Alexa Fluor647-positive cells proportion of mCherry/GFP double positive (DP) hPGCLCs (*n* = 2). *, *P* < 0.05. Two-tailed Student’s *t*-test. (G) Graphical summary illustrating the truncated DAZL disrupted its interaction with PABP and diminished the expression of NANOS3 protein, thereby increased the level of apoptosis in PGCs.

It has been previously reported that *Nanos3* plays a vital role in suppressing the Bax-dependent apoptotic pathway, and *Nanos3* knockout mice exhibit severe loss of germ cells due to the elimination of the migrating PGCs [9]. Considering the downregulation of NANOS3 expression in the truncated DAZL hPGCLCs, we further investigated the impact of the truncated DAZL on cell apoptosis using Annexin V assay. Four-day hPGCLCs overexpressing WT DAZL or truncated DAZL were subjected to Annexin V assay. The results showed a significantly higher proportion of Annexin V+ apoptotic cells in the truncated DAZL hPGCLCs compared to the WT groups, indicating that the truncated DAZL promoted cell apoptosis during hPGCLCs differentiation (Fig. 2C and 2D). Given the role of *Nanos3* in suppression mouse hPGCs apoptosis pathway, we further investigated the effect of *NANOS3* knockdown on apoptosis in wildtype hPGCLCs. hESCs were co-transduced with NANOS3-eGFP silencing lentivirus and mCherry-WT DAZL overexpressing lentivirus, followed by 4-day differentiation. The eGFP^+^/mCherry^+^ double positive cells were sorted to analyze the proportion of Annexin V−APC+ cells. The results revealed that *NANOS3* knockdown significantly enhanced cell apoptosis in wildtype hPGCLCs (Fig. 2E and 2F), indicating that the down-regulation of NANOS3 contributed to increased cell apoptosis in c.808C>T hPGCLCs.

In this study, we have identified a novel homozygous point mutation, c.808C>T, in the *DAZL* gene of a patient with POI, which causes truncation at the C-terminal end of DAZL. Molecular and cellular experiments have elucidated the truncated DAZL disrupted its interaction with PABP and diminished the expression of NANOS3 protein. The lower level of NANOS3 increased apoptosis in PGCs (Fig. 2G). This study enhances our understanding of the molecular mechanisms underlying POI development and highlights the potential therapeutic implications of modulating DAZL and NANOS3 expression in patients suffering POI.

## Research limitations

Although our study revealed the translational regulatory defects of truncated DAZL on its target mRNAs during hPGCLCs differentiation, we were unable to verify the NANOS3 expression pattern in the PGCs of POI patient. This was due to the challenges in obtaining clinical samples through invasive procedures. If PGCs can be obtained from the POI patient, single-cell transcriptome and translatome analysis can be used to examine the expression profiles of the samples [10].

## Supplementary Material

lnae007_suppl_Supplementary_Tables_S1_Figures_S1
