# Supplementary material for: Truncated DAZL mutation reduces NANOS3 expression in primordial germ cells and leads to premature ovarian insufficiency
Source: Life Med. 2024 Feb 9;3(2):lnae007. doi: 10.1093/lifemedi/lnae007 (PMC11749114; doi:10.1093/lifemedi/lnae007)
Supplement: lnae007_suppl_Supplementary_Tables_S1_Figures_S1 [file lnae007_suppl_Supplementary_Tables_S1_Figures_S1.docx]

**Truncated DAZL mutation reduces NANOS3 expression in primordial germ cells and leads to premature ovarian insufficiency**

Supplementary Methods

*Patient*

A 27-year-old woman was referred with the history of secondary amenorrhea and 4-year infertility. She had received estro-progestin treatment to set up artificial menstrual cycle since the age of 25. Physical examination revealed that she presented with no somatic anomalies or mental retardation. Transvaginal ultrasonography revealed vaguely visible bilateral ovaries and relatively small uterus measuring 4.1×3.2 cm. The laboratory analysis showed that her karyotype was 46,XX and serum levels of follicle stimulating hormone (FSH), luteinizing hormone (LH) and anti-Müllerian hormone (AMH) were 22.30 IU/L, 7.85 IU/L and < 0.08 ng/mL respectively. The patient had no sibling and her mother still reported regular menstruation. The patient had signed the informed consent.

*Cell lines*

The hESC line H9 (purchased from WiCell, Inc.) was maintained on 1% Matrigel (Corning,354248)-coated 6-well plate, cultured in hPSC-CDM (Cauliscell, 400105) supplemented with hPSC-CDM supplement. The HEK293FT cell line (Invitrogen) was cultured in 90% DMEM (Corning, 10017-CV) supplemented with 10% fetal bovine serum (GEMINI, #900-108), 1% GlutaMax (Thermo Fisher Scientific, 35050061), 1% MEM NEAA (Thermo Fisher Scientific, 11140050), 0.5 mg/mL geneticin (Millipore, 108321422) and 1% Penicillin-Streptomycin (Thermo Fisher Scientific, 15140122). All cell lines were cultured at 37°C with 5% CO_2_ and were tested negative for mycoplasma.

*Vectors construction and lentivirus production*

The human wildtype *DAZL* CDS and c.808C > T *DAZL* variant were subcloned into pENTR-D-TOPO plasmids to create D-TOPO-mCherry-P2A-3×Flag-wildtype DAZL and c.808C > T DAZL vectors. These entry vectors carrying wildtype or c.808C > T DAZL were recombined with p2k7 plasmids using Gateway LR Clonase II Enzyme Mix (Invitrogen, 11791020) to generate the lentiviral vectors. These vectors were transfected into HEK293FT cells with Vsvg and Δ8.9 plasmids to produce lentivirus as previously described (Yan, A., et al. 2022).

*Knockdown of NANOS3*

The small-hairpin RNA (shRNA) targeting human NANOS3 was designed from GPP Web Portal (portals.broadinstitute.org/gpp/public) and the shRNA sequence was listed in Table S2. The H1-eGFP plasmid was linearized and ligated with annealed primers to generate NANOS3 silencing (shNANOS3) vector. The H1-eGFP plasmid with LacZ silencing sequence was used as negative control (sequence listed in Table S2). For silencing of NANOS3, hESCs were transduced with lentivirus overexpressing wildtype DAZL and lentivirus carrying shNANOS3-eGFP or shLacZ-eGFP, followed by 4-day differentiation. Cells were subjected to flow cytometry to isolate mCherry/eGFP double positive cells for subsequent apoptosis analysis.

*Derivation of human PGCLCs from hESCs*

For derivation of human PGCLCs, hESCs were digested by EDTA solution (Cellapy, CA3001500), seeded onto a plate pretreated with Matrigel and were cultured in hESCs conditional medium for 1 day recovery. hESCs medium consisted of 80% DMEM and 20% KnockOut SR Medium (Thermo Fisher Scientific, 10828028) supplemented with 8ng/mL basic fibroblast growth factor (R&D Systems, 3718-FB-010), 1% GlutaMax, 1% MEM NEAA and 1% Penicillin-Streptomycin. The inactivated mouse embryo fibroblasts (MEFs) were cultured in hESCs medium for 1 day and the supernatants were harvested as hESCs conditional medium. The hESCs differentiation medium consisted of 90% KnockOut DMEM (Thermo Fisher Scientific, 10829018) and 10% fetal bovine serum supplemented with 1% GlutaMax, 1% MEM NEAA and 1% Penicillin-Streptomycin. On the first day, marked as day 0, hESCs were cultured in hESCs differentiation medium and induced with 50 ng/mL BMP4 (R&D) and 50 ng/mL BMP8a (R&D) for 2 h. After 2h of inducement, cells were transduced with lentivirus and differentiated in hESCs differentiation medium with 50 ng/mL BMP4 and 50 ng/mL BMP8a for another 4 or 6 days.

*Dual-luciferase reporter assay*

The 3'UTR sequences of human NANOS3, VASA and SYCP3 were downloaded from NCBI website (www.ncbi.nlm.nih.gov), and were cloned into the psiCHECK2 vector carrying renilla/firefly-dual luciferase reporter (Promega, C8021). HEK293FT cells were transfected with psiCHECK2 luciferase vectors carrying NANOS3, VASA or SYCP3 3'UTR and p2k7 vectors overexpressing either wildtype or c.808C > T DAZL using VigoFect (Vigorous) in replications of four. The empty psiCHECK2 and p2k7 vectors were used as negative controls. The relative luciferase activities were measured as previously described (Jung, D., et al. 2017).

*Western blot analysis*

The hPGCLCs were lysed in ice-cold RIPA buffer (50 mM Tris, 150 mM NaCl, 0.5% Sodium deoxycholate, 1% NP-40, 0.1% SDS, pH = 8) containing protease inhibitor (Roche, 04693132001). The lysates were purified by centrifugation at 12,000 r/min for 20 min at 4°C. Protein samples were separated by 12% SDS-PAGE and were transferred to the PVDF membrane (Millipore). Antibodies were diluted in TBST containing 5% non-fat milk (BD Biosciences, 232100). Targeted proteins were immunoblotted with primary antibodies at 4°C overnight, followed by incubation with secondary antibodies for 1 h at room temperature. The bands were visualized by chemiluminescence. Antibodies were listed in Table S3.

*Co-immunoprecipitation*

HEK293FT cells were transfected with wildtype DAZL or c.808C > T DAZL for 48h, followed by flow cytometry to sort 2×10^6^ mCherry-DAZL positive cells. Cells were washed with cold DPBS for 3 times and lysed in IP lysis buffer (50 mM Tris–HCl, pH = 7.4, 150 mM NaCl, 10% glycerol, 1% TritonX-100) supplemented with protease inhibitor for 30 min at 4°C. The cell lysates were purified by centrifugation at 13,500 rpm for 15 min at 4°C. Supernatants were incubated with primary antibodies bound on the Protein G magnetic beads (Thermo Fisher Scientific, 10003D) at 4°C overnight. 2% of cell lysate was retained as input. Immunocomplexes were washed 3 times with IP washing buffer (50 mM Tris–HCl, pH = 7.4, 500 mM NaCl, 10% glycerol, 0.5% TritonX-100) and were eluted for subsequent western blot analysis. Antibodies were listed in Table S3.

*RNA extraction and real-time quantitative PCR*

Total RNA was extracted from cells using TRIzol Reagent (Thermo Fisher Scientific, 15596026) according to its manufacturer’s protocol. Reverse transcription was performed using TransScript® II One-Step gDNA Removal and cDNA Synthesis SuperMix (Transgen, M20105) as the manufacturer’s standard protocol. cDNA was subjected to quantitative PCR amplification with primers for GAPDH and DAZL using ChamQ Universal SYBR qPCR Master Mix (Vazyme, Q711-02). The samples were run in three technical replicates. The sequences of primers were listed in Table S4 and S5.

*Apoptosis assay and flow cytometry*

During 4-day differentiation of hPGCLCs, the viral supernatants were replaced by fresh hESC differentiation medium with 50 ng/mL BMP4 and 50 ng/mL BMP8a on the day after transfection. Instead of changing medium, freshly prepared hESC differentiation medium was added to each well every day. After 4-day differentiation, both suspended and attached hPGCLCs were collected in cold DPBS and incubated with Alexa Fluor488-conjugated Annexin V (Solarbio, CA1040) or Alexa Fluor647-conjugated Annexin V (Solarbio, CA1050) for 5 min at room temperature (5 μL Annexin V/100 μL binding buffer), protected from light. Cells were subjected to flow cytometry and the data were analyzed using the FlowJo software.

*RNA-seq*

After 4-day or 6-day differentiation of hPGCLCs, mCherry-wildtype DAZL or mCherry-c.808C > T DAZL positive cells were sorted by flow cytometry. Total RNA was extracted from sorted cells using TRIzol Reagent and was subjected to quantification using the Qubit 2.0 Fluorometer (Life Technologies). The quality of RNA samples was examined using the Agilent 2100 Bioanalyzer. Purification of RNA samples was performed by oligo-attached poly-T magnetic beads. Library was constructed using Illumina TruSeq RNA Library Prep Kit according to the manufacturer’s instruction. Samples were loaded on the Illumina HiSeq2000 instrument for paired-end sequencing.

*Analysis of RNA-Seq data*

Raw sequences were analyzed using FastQC (v0.11.8) for quality control. Trim-galore (v0.6.4) was used to remove adaptor sequences. The filtered reads were mapped to human genome 38 using hisat2 (v2.2.0). Stringtie (v2.1.4) was used to calculate read counts. The differentially expressed genes were identified using DESeq2 (FPKM median > 1, Fold Change ≥ 1, *P*-value ≤ 0.05).

*Statistical analysis*

The data were presented as mean ± SD, and *P* < 0.05 was considered to be statistically significant. Statistical evaluation was performed with GraphPad Prism 8 (GraphPad software). Statistical significances of data were calculated using unpaired Student’s *t* test and one-way analysis of variance (ANOVA).

Supplementary Materials

Table S1. Information of c.808C > T DAZL in primary ovarian insufficiency patient

^a^gnomAD (www.gnomad-sg.org): The allele frequency indicates variants polymorphism.

^b^Mutation taster (www.mutationtaster.org): The probability value indicates confidence level of prediction.

^c^Cadd (Combined Annotation-Dependent Depletion): variant deleteriousness prediction tool with high score indicating disease causing.

^d^Dann (Deleterious Annotation of genetic variants using Neural Networks): The prediction scores vary between 0 to 1 with high score indicating damaging.

Table S2. The oligonucleotides for shRNA-mediated NANOS3 knockdown

| shRNA | Sequence |
| --- | --- |
| shNANOS3 | GCCTGTGCTCTTTCTGCAAAC |
| shLacZ | AAATCGCTGATTTGTGTAGTC |

Table S3. The antibodies for Western blot and co-immunoprecipitation

| Antibodies | Lot Number | Company |
| --- | --- | --- |
| Monoclonal anti-Flag M2 antibody | F1804 | Sigma-Aladrich |
| Polyclonal anti-NANOS3 antibody | Ab70001 | Abcam |
| Polyclonal anti-PABP antibody | Ab21060 | Abcam |
| Monoclonal anti-alpha Tubulin antibody  Polyclonal anti-IgG antibody | Ab7291  B30010 | Abcam  Abmart |

Table S4. The oligonucleotides for RT-qPCR

| Gene | Forward Primer | Reverse Primer |
| --- | --- | --- |
| *DAZL* | CAGTGGCCTGTTGGGGAGCAA | GTGGGCCATTTCCAGAGGGTGG |
| *GAPDH* | TGTTGCCATCAATGACCCCTT | CTCCACGACGTACTCAGCG |

Table S5. The PCR conditions

| Cycle (*n*) | Temperature (°C) | Time |
| --- | --- | --- |
| 1 | 95°C | 2 min |
| 2–34 | 95°C  *T*_m_−5°C | 20 s  20 s |
|  | 72°C | 4 kb/min |
| 33 | 72°C | 5 min |


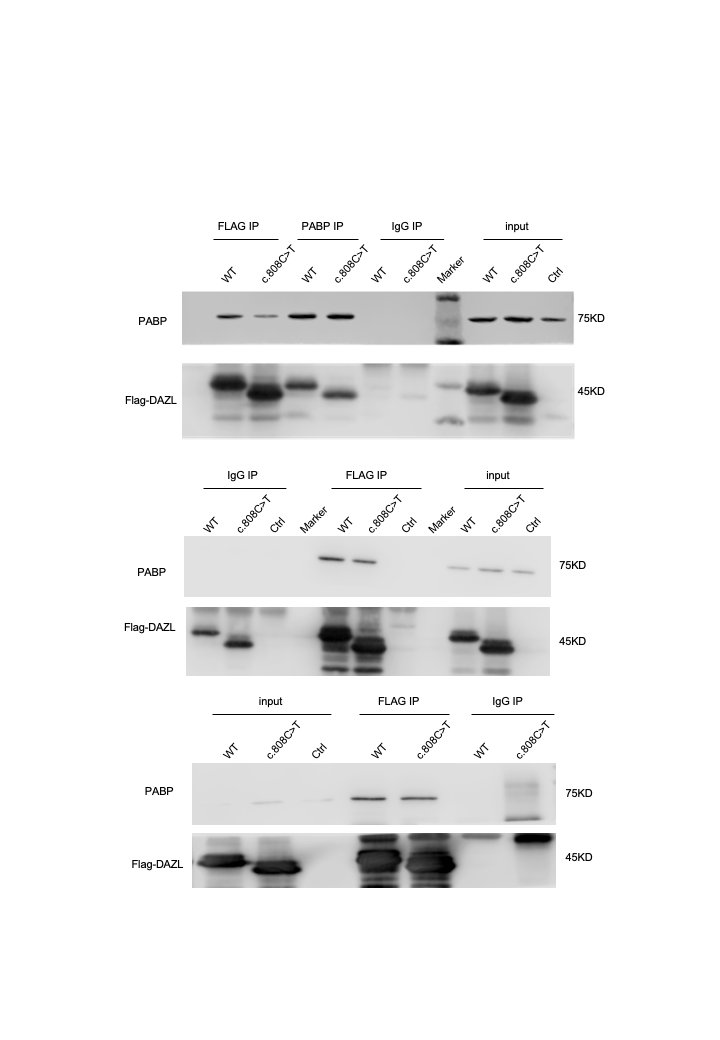


Figure S1. The interaction between PABP and WT or c.808C > T DAZL were validated by co-immunoprecipitation assays. Samples were obtained by Flag pull-down (Flag IP) and PABP pull-down (PABP IP). Mouse IgG was used as negative control (IgG IP). Three independent co-IP experiments were shown.
